# The secretome of induced pluripotent stem cells reduces lung fibrosis in part by hepatocyte growth factor

**DOI:** 10.1186/scrt513

**Published:** 2014-11-10

**Authors:** Amiq Gazdhar, Iwona Grad, Luca Tamò, Mathias Gugger, Anis Feki, Thomas Geiser

**Affiliations:** Department of Pulmonary Medicine, University Hospital, Bern, Friburgstrasse, Bern, 3010 Switzerland; Department of Clinical Research, University of Bern, Murtenstrasse 50, Bern, 3008 Switzerland; Stem Cell Laboratory, Geneva University Hospital, Rue Gabrielle-Perret-Gentil 4, Geneva, 1205 Switzerland; Graduate School of Biomedical Science, University of Bern, Freierstrasse 1, 3012 Bern, Switzerland; Institute of Pathology, University of Bern, Murtenstrasse 31, Bern, 3010 Switzerland; Department of Obstetrics and Gynaecology, Freiburg Hospital, PO Box 1708, Freiburg, Switzerland

## Abstract

**Introduction:**

Idiopathic pulmonary fibrosis (IPF) is a progressive and irreversible fibrotic lung disease, resulting in respiratory insufficiency and reduced survival. Pulmonary fibrosis is a result of repeated alveolar epithelial microinjuries, followed by abnormal regeneration and repair processes in the lung. Recently, stem cells and their secretome have been investigated as a novel therapeutic approach in pulmonary fibrosis. We evaluated the potential of induced pluripotent stem cells (iPSC) conditioned media (iPSC-cm) to regenerate and repair the alveolar epithelium *in vitro* and improve bleomycin induced lung injury *in vivo*.

**Methods:**

IPSC-cm was collected from cultured iPSC derived from human foreskin fibroblasts and its biological effects on alveolar epithelial wound repair was studied in an alveolar wound healing assay *in vitro*. Furthermore, iPSC-cm was intratracheally instilled 7 days after bleomycin induced injury in the rat lungs and histologically and biochemically assessed 7 days after instillation.

**Results:**

iPSC-cm increased alveolar epithelial wound repair *in vitro* compared with medium control. Intratracheal instillation of iPSC-cm in bleomycin-injured lungs reduced the collagen content and improved lung fibrosis in the rat lung *in vivo*. Profibrotic TGFbeta1 and α-smooth muscle actin (α-sma) expression were markedly reduced in the iPSC-cm treated group compared with control. Antifibrotic hepatocyte growth factor (HGF) was detected in iPSC-cm in biologically relevant levels, and specific inhibition of HGF in iPSC-cm attenuated the antifibrotic effect of iPSC-cm, indicating a central role of HGF in iPSC-cm.

**Conclusion:**

iPSC-cm increased alveolar epithelial wound repair *in vitro* and attenuated bleomycin induced fibrosis *in vivo*, partially due to the presence of HGF and may represent a promising novel, cell free therapeutic option against lung injury and fibrosis.

**Electronic supplementary material:**

The online version of this article (doi:10.1186/scrt513) contains supplementary material, which is available to authorized users.

## Introduction

Idiopathic pulmonary fibrosis (IPF) is characterized by a chronic progressive scaring in the lung mainly due to improper alveolar wound healing and failure of the alveolar epithelium to re-epithelialize in response to epithelial injury [1–3]. Although IPF is a devastating disease of unknown etiology, there is currently no treatment available to reverse lung destruction and induce alveolar epithelial regeneration. Most approaches were developed towards slowing the worsening of the disease using anti-inflammatory or antifibrotic agents, but with little success.

Cell-based approaches have been proposed recently as new possibilities to regenerate various organs [4–7], including the fibrotic lung [8, 9]. This usually implies administration of exogenously derived stem cells that support regeneration and modulate the progress of the disease. Bone marrow-derived stromal cells have been tested in many laboratory disease models [10] and also in clinical trials [11, 12]. Although the isolation and expansion of these cells is well established, there are some concerns with regards to their efficiency and availability, particularly in older patients [13]. Another obvious source is embryonic stem cells, but the complexity of the derivation technique and ethical issues have hindered advancement in clinical applications. The generation of human induced pluripotent stem cells (iPSCs) has opened a new avenue for patient-specific cell-based therapies [14]. Mouse [15] and human iPSCs have been derived with characteristics of embryonic stem cells, including pluripotency and ability to differentiate and contribute to the three germ layers and thus the potential to differentiate into any cell type [16]. Recently iPSCs have been successfully used for regeneration in spinal injury, cerebral vascular injury and acute lung injury in animal models [17–19], providing evidence that iPSCs could be very promising candidates for cell-based therapies in a variety of diseases.

In clinical settings, direct use of cells is a complex process and the fate of administered iPSCs in the human body is not clear. Also, the effect of pluripotency genes and the transcriptional memory of the somatic cells that the reprogrammed cells might carry are still not fully understood [20]. Cell-free methods carrying a similar therapeutic potential could therefore offer an obvious advantage for a clinical translation in the future. One of these possibilities is the use of cell-conditioned media obtained from stem cells, since released factors from stem cells normally induce biological responses similar to the cells when tested *in vitro* or *in vivo*. However, the characteristics of induced pluripotent stem cell conditioned medium (iPSC-cm), including analysis of the iPSC-cm secretome, and its potential benefits have not yet been identified in detail.

In the present study we show that iPSC-cm may be a novel and promising source of cell-based yet cell-free therapy for pulmonary fibrosis, since iPSC-cm induces alveolar epithelial repair i*n vitro* and reduces lung fibrosis in a bleomycin-induced animal model *in vivo*, at least in part by a hepatocyte growth factor (HGF)-dependent mechanism.

## Materials and methods

### Generation of induced pluripotent stem cells

#### Ethical approval

In this study iPSCs were generated using commercially available cell lines of human foreskin fibroblasts. No material from patients or healthy individuals was used and therefore written or verbal consent was not required, therefore no ethical approval was needed for this study.

Human foreskin fibroblasts (line CRL-2429; American Type Culture Collection (ATCC), Rockville, MD, USA) were used to generate iPSCs as described elsewhere [21, 22]. After expansion, 5 × 10^4^ human fibroblasts were infected with multiplicity of infection of 5 pSin-EF2-Nanog-Pur. The cells were treated with puromycin for 5 days and then retransduced with three viruses: pSin-EF2-Sox2-Pur, pSin-EF2-Oct4-Pur and pSin-EF2-Lin28-Pur (multiplicity of infection 5). iPSCs were maintained on irradiated human foreskin fibroblasts (CCD1; ATCC) in knockout Dulbecco’s modified Eagle’s medium supplemented with 20% serum replacement, 2 mmol/l Glutamax, 40 μg/ml gentamycin and 100 μmol/l β-mercaptoethanol (all Gibco, 111 Grand Island, NY, USA) in the presence of 100 ng/ml β-fibroblast growth factor (bFGF; Peprotech London, UK) and were passaged mechanically every 2 or 3 days.

### Embryoid body formation and *in vitro*differentiation

Cells were differentiated *in vitro* to examine whether derived iPSCs are able to differentiate into multilineage cell types. Embryoid bodies were formed after culturing in suspension in knockout Dulbecco’s modified Eagle’s medium supplemented with 10% normal calf serum, 1 mM l-glutamine, 100 μM nonessential amino acids, 100 μM 2-mercaptoethanol, 50 U/ml penicillin and 50 mg/ml streptomycin (Gibco/Invitrogen) for 7 days, and then transferred to gelatin-coated dishes. After 14 days, differentiated cells were examined by immunostaining as described below.

### Immunohistochemistry

To confirm pluripotency, the newly generated colonies were immunostained with OCT3/4 (1:50), NANOG (1:50), SSEA4 (1:50), and TRA-1-81 (1:200) (Santa Cruz Biotechnologies, Dallas, Texas, USA) at the given concentrations. The colonies were fixed in 3% paraformaldehyde for 30 minutes, washed with phosphate-buffered saline (PBS) and permeabilized with PBS and 0.5% Triton. After blocking with 5% bovine serum albumin, the colonies were incubated with primary antibodies at appropriate concentrations overnight, followed by appropriate secondary antibody treatment. For multilineage cell staining, the cells were fixed with 3% paraformaldehyde and permeabilized with 0.5% Triton. After blocking with bovine serum albumin, the cells were incubated overnight with β-tubulin III (ectoderm marker, 1:50), nestin (endoderm marker, 1:50) and alpha smooth muscle actin (αSMA; mesoderm marker, 1:50) (Santa Cruz Biotechnologies), followed by appropriate secondary antibody treatment. The results were evaluated using a Leica Fluroscence DMI 4000-B (Leica Microsystems Heerbrugg, St Gallen, Switzerland).

### Induced pluripotent stem cell conditioned medium

Ten to 12 iPSC colonies (5.05 ± 0.65 × 10^5^ live cells) were grown in knockout media without serum replacement and without bFGF on plates coated with Cell Start (Gibco/Invitrogen) (feeder-free plate) for 24 hours. The iPSC-cm was collected, centrifuged and further used for experiments. Knockout media without serum replacement and without bFGF was used as negative control. Similarly, the conditioned media from CCD1 human foreskin fibroblasts (ATCC) was used as control conditioned media.

### Annexin–propidium iodide staining for the live/dead cell ratio

Propidium iodide (PI; Invitrogen, Lucerne, Switzerland) and Annexin V-Alexa647 (BioLegend, Lucerne, Switzerland) staining was performed to measure cell death and apoptosis, respectively. The iPSC colonies growing on Cell Start coated plates were trypsinized at room temperature for 5 minutes and the cells were suspended in PBS^–/–^ (no calcium, no magnesium; Invitrogen, Grand Island, NY, USA). The cells were incubated with Annexin V-Alexa647 antibodies (1:50) for 30 minutes. PI was added just before measurement (1:100). Cells were analyzed by flow cytometry using an LSRII flow cytometer (BD Biosciences, Franklin lakes, NJ, USA).

### *In vitro*alveolar epithelial wound repair assay

Human A549 alveolar epithelial-like cells (ATCC) were cultured to confluence in a 24-well plate using RPMI 1640 containing 10% fetal bovine serum (Gibco). The confluent epithelial monolayer was mechanically wounded with a pipette tip and further cultured in either iPSC-cm or control media. Images of the wound surface were captured at time 0 and after 24 hours using an inverted Leica DMI4000 B microscope. Quantification of the wound surfaces was performed using Image J image software (National institute of health, Bethesda, Maryland, USA), and alveolar epithelial wound repair was expressed as the percentage of epithelial wound closure after 24 hours.

### Animals

Male Fisher F344 rats (240 to 280 g) were obtained from Charles River Laboratories GmbH (Sulzfeld, Germany). Experiments were performed in accordance with the standards of the European Convention of Animal Care. The study protocol was approved by the University of Bern Animal Study Committee.

### Instillation of bleomycin

At day 0 of the protocol, F344 rats (220 to 240 g) were anesthetized by inhalation of 4% isoflurane in a glass chamber, intubated with a 14 GA intravenous catheter (Insyte, Madrid, Spain) and instilled intratracheally with bleomycin (1.28 U/rat) to both lungs. The dosage of bleomycin was based on preliminary experiments showing induction of pulmonary fibrosis with lowest mortality.

### Instillation of iPSC-cm and control media

Seven days after intratracheal instillation of bleomycin (1.28 U/rat in both lungs), the animals were anesthetized as explained above and divided into three groups with seven in each group. The first group was treated by intratracheal administration of knockout media alone 500 μl (media control), while in the second group 500 μl iPSC-cm was intratracheally administered and in the third group 500 μl conditioned media obtained from fibroblasts (CCD1) was instilled as control.

### Determination of HGF levels and HGF inhibition experiments

iPSC-cm was collected and HGF levels were measured using the Bio-Plex suspension array system (Bio-Rad Laboratories, Basel, Switzerland), according to the manufacturer’s instructions.

To study possible mechanisms of iPSC-cm effects on alveolar epithelial repair *in vitro* and lung fibrosis *in vivo*, we focused on the role of HGF due to its antifibrotic activities. iPSC-cm was incubated with human HGF neutralizing antibodies (R&D Systems, Abingdon, UK) for 30 minutes at 37°C. For *in vitro* experiments, iPSC-cm was incubated with HGF antibodies at different concentrations (0.01, 0.1, and 0.8 ng/ml, maximal dose as recommended by the manufacturer). For *in vivo* experiments, a dose of 8 μg/ml HGF antibodies was used.

We instilled iPSC-cm treated with HGF neutralizing antibodies intratracheally in rats 7 days after bleomycin-induced lung injury with a volume of 500 μl (*n* =5). As controls, we instilled HGF neutralizing antibodies alone dissolved in the same volume of buffer (*n* =3). All animals were sacrificed 7 days after treatment.

### Assessment

At day 14 (7 days after iPSC-cm instillation) animals were anesthetized as described above. Thiopental (50 mg/kg body weight) was administered intraperitoneally. The heart–lung block was explanted and tissue samples were collected for further analysis.

### Histology

Routine hematoxylin and eosin staining was performed with formalin-fixed tissue sections. To evaluate the extent of pulmonary fibrosis, the scoring system of Ashcroft and colleagues [23] was used by a trained pathologist as reported previously [24].

### Collagen assay

The level of acid-soluble collagen in lung tissue was determined with a Sircol collagen assay (Biocolor Ltd, County Antrim, UK) according to the manufacturer’s instructions. Briefly, the lungs were excised and snap frozen after having measured the wet weight. The frozen lungs where homogenized in 1× PBS. The homogenate was treated with Sircol dye reagent for 30 minutes at room temperature with shaking. After brief centrifugation, the pellet was dissolved in alkali reagent and was measured at 540 nm.

### Real-time polymerase chain reaction measurement of transforming growth factor beta expression levels

Quantitative polymerase chain reaction analysis was carried out to evaluate relative mRNA level changes of transforming growth factor beta 1 (TGFβ1) in different groups. Total RNA was isolated from the lung using Trizole (Life Science, Grand Island, NY, USA) following the manufacturer’s instructions. First-strand cDNA was synthesized using the Omniscript RT kit (Qiagen, Duesseldorf, Germany). The reaction was performed using the Rat TGFβ1 Taqman gene expression assay (Applied Biosystems, Life Technologies, Grand Island, NY, USA) on 7500 Fast (Applied Bioscience) and the 18s gene was used as a housekeeping gene (Applied Biosystems, Life Technologies, Zug, Switzerland). The relative gene expression changes were calculated using the ΔΔCt method, and the regulation factor was calculated as RF =2 − ΔΔCt.

### Immunohistochemistry for αSMA in paraffin-embedded lung tissue

The formalin-fixed sections were deparaffinized in a xylene series and rehydrated through a decreasing ethanol series. The slides were pretreated by microwave in citrate buffer (100 mM, pH 7.0) for 10 minutes, washed three times with 1× Tris-buffered saline +0.1% Tween and incubated overnight at 4°C in αSMA (1:100; Sigma Aldrich, St. Louis, MO, USA), and stained with EnVision + ™ System–horseradish peroxidase–3,3′-diaminobenzidine (Dako, Baar, Switzerland). The positive signals are brown stained by 3,3′-diaminobenzidine.

## Results

### Generation and characterization of iPSCs

**Figure 1:**
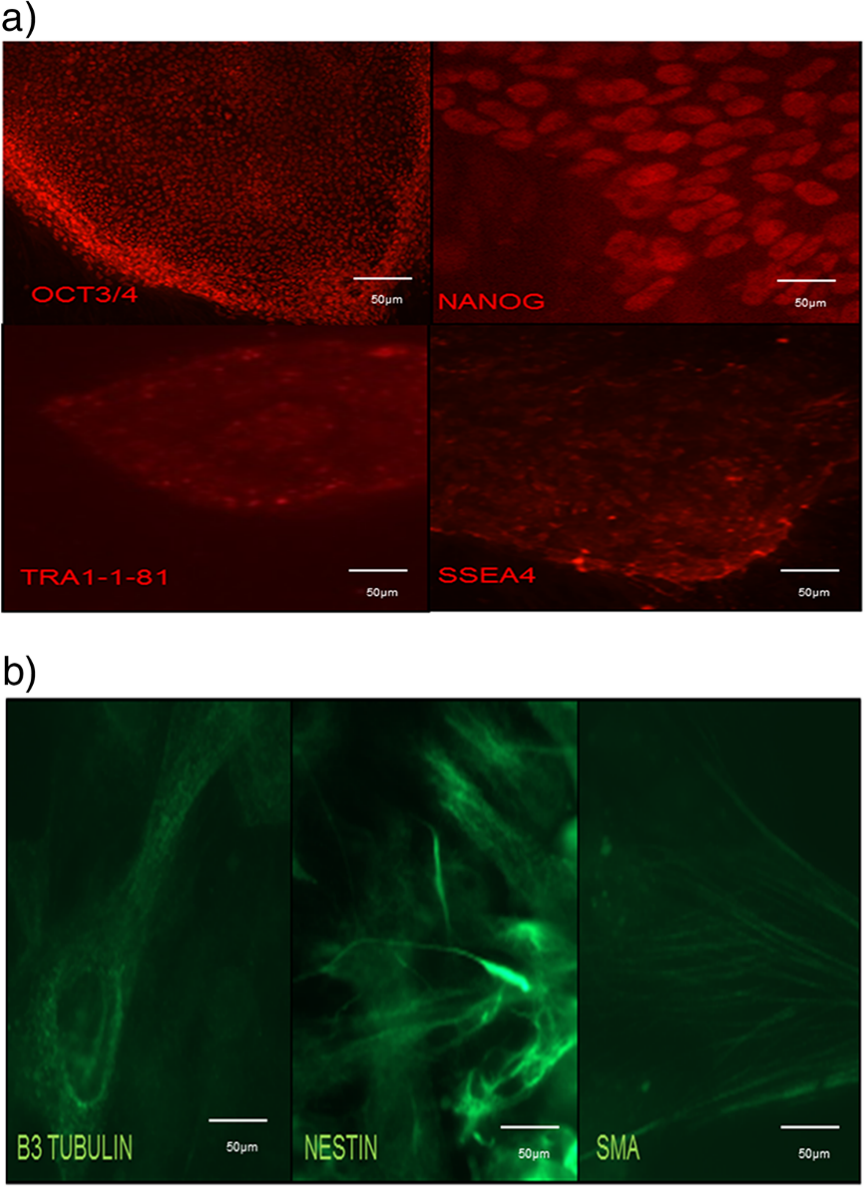
**Immunophenotypic profiles of induced pluripotent stem cell colonies and differentiation of embryoid bodies. (a)** Following viral transfection of fibroblasts and culture for 2 to 3 weeks in embryonic stem cell culture medium, induced pluripotent stem cell (iPSC) colonies stained positive for the pluripotent markers OCT4, Nanog, SSEA4 and TRA-1-81 respectively. **(b)** Derived iPSCs have the ability to differentiate into the three germ layers *in vitro* as shown after *in vitro* differentiation of embryoid bodies: β-tubulin III (ectoderm marker), nestin (endoderm marker) and smooth muscle actin (SMA; mesoderm marker).

After viral transfection of the human foreskin fibroblast with vectors carrying the four reprogramming genes, iPSC colonies were detected after 2 to 3 weeks in culture. These colonies stained positive for markers of pluripotency, in particular OCT3/4, NANOG, SSEA4 and TRA-1-81 (Figure 1a).

Embryoid bodies were formed after culturing iPSCs in suspension for 7 days and subsequent plating for 2 weeks. Differentiated cells expressing β-tubulin III (ectoderm marker), nestin (endoderm marker) and αSMA (mesoderm marker) were detected, indicating that the iPSCs are pluripotent and can be differentiated into cells of ectoderm, endoderm and mesoderm lineages (Figure 1b).

### Induced pluripotent stem cells grow well during preparation of conditioned media (in media without serum replacement and bFGF)

Annexin–PI staining of the iPSCs growing in serum replacement and bFGF-free media revealed no drastic cell death. The percentage of PI-positive and Annexin-positive events was 28.2% for PI and 24.9% for Annexin in cells growing in media without serum replacement and bFGF compared with 27.2% for PI and 25.1% Annexin in cells growing in normal media (Additional file 1).

### Induced pluripotent stem cell conditioned medium increases alveolar epithelial wound repair *in vitro*

**Figure 2:**
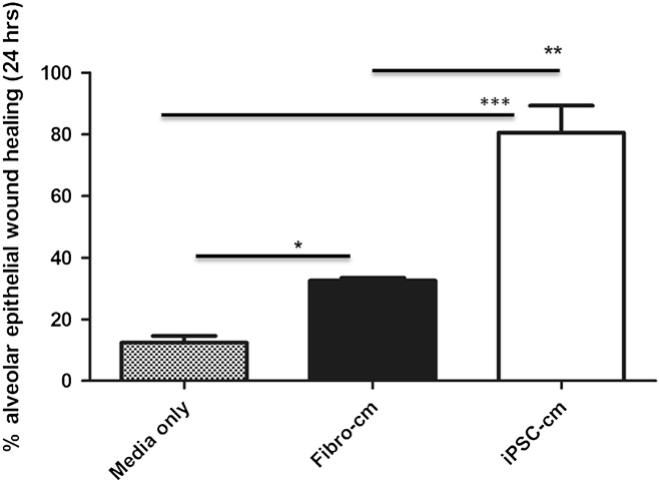
**Induced pluripotent stem cell conditioned medium improves alveolar epithelial wound repair** ***in vitro*** **.** Alveolar epithelial wound repair *in vitro* was increased after treatment of the wounded A549 alveolar epithelial monolayer with induced pluripotent stem cell conditioned medium (iPSC-cm) compared with fibroblast (CCD1) conditioned medium (Fibro-cm) and serum-free media as control. Data indicated as mean ± standard error of the mean. *P <0.001, **P <0.001,***P <0.0001, respectively.

Wounded monolayers of A549 alveolar epithelial cells treated with iPSC-cm showed markedly improved alveolar epithelial wound repair *in vitro* (80.61 ± 8.74% epithelial wound healing after 24 hours as compared with wounded A549 cells treated with fibroblast conditioned medium (CCD1-cm; 30.10 ± 2.96%) and serum-free control medium (12.39 ± 2.21%, *P* <0.001)), indicating that iPSC-cm contains biologically active mediators that induce alveolar epithelial repair *in vitro* to a significantly higher degree than CCD1-cm or serum-free medium control (Figure 2).

### Induced pluripotent stem cell conditioned medium reduces fibrosis in bleomycin-injured rat lungs *in vivo*

**Figure 3:**
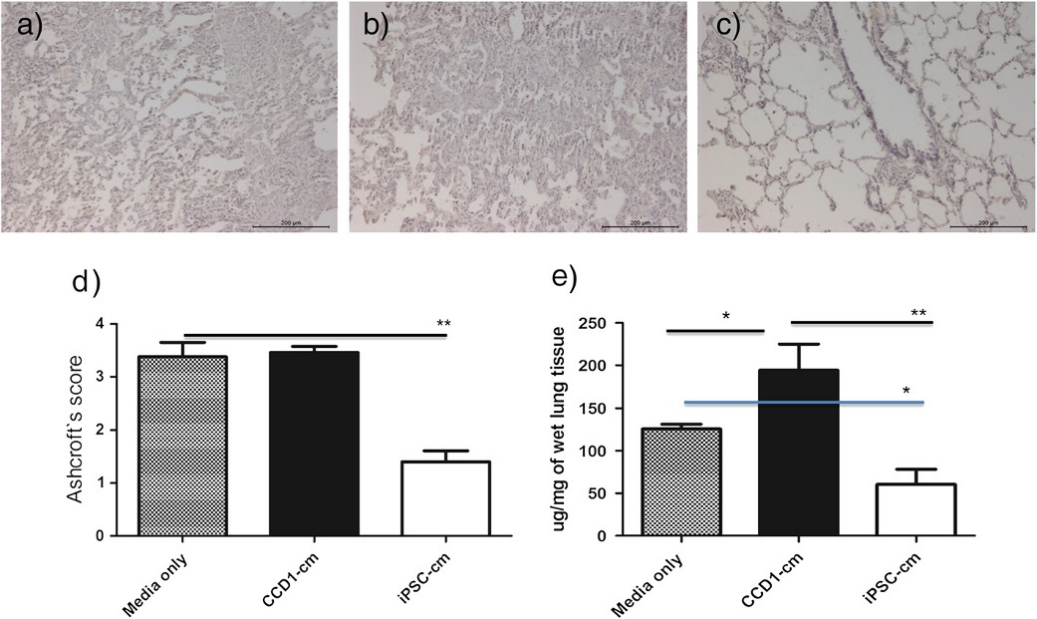
**Histological and biochemical analysis of lung fibrosis** ***in vivo*** **.** Histological analysis of injured lungs treated with different types of conditioned media. Marked fibrosis was seen in the control groups treated with **(a)** media only **(b)** or fibroblast conditioned medium (CCD1-cm), whereas a significant decrease of fibrosis was observed in **(c)** the induced pluripotent stem cell conditioned medium **(**iPSC-cm)-treated group. Hematoxylin and eosin staining, scale bar =200 μm. **(d)** Semiquantitative histological analysis of lung fibrosis using the Ashcroft score showed significant reduction of fibrosis after treatment with iPSC-cm compared with media control or CCD-1-cm. Data indicated as mean ± standard error of the mean (SEM), **P <0.001. **(e)** In accordance with histological data, the collagen content was reduced after treatment with iPSC-cm compared with media control or CCD-1-cm. Data indicated as mean ± SEM, *P <0.01, **P <0.001.

Administration of iPSC-cm to bleomycin-injured rat lungs showed marked improvement of fibrosis as assessed by histology (Figure 3c) compared with CCD1-cm (Figure 3b) or serum-free media control (Figure 3a). Semi-quantitative analysis was performed using the Ashcroft score (1.40 ± 0.24 (iPSC-cm) vs. 3.47 ± 0.16 (CCD1-cm) and 3.4 ± 0.46 (control media), *P* <0.001) (Figure 3d). In accordance, a marked reduction in the soluble collagen content was detected in the lungs of iPSC-cm-treated animals compared with animals treated with CCD1-cm or serum-free media control (60.46 ± 15.39 μg/mg vs. 176.4 ± 49.86 μg/mg (CCD1-cm) and 125.8 ± 5.4 μg/mg wet lung (media control), *P* <0.001) (Figure 3e), indicating antifibrotic activities of iPSC-cm in the *in vivo* bleomycin lung injury model.

### HGF secreted by iPSCs markedly contributes to alveolar epithelial repair *in vitro*and reduces pulmonary fibrosis *in vivo*

**Figure 4:**
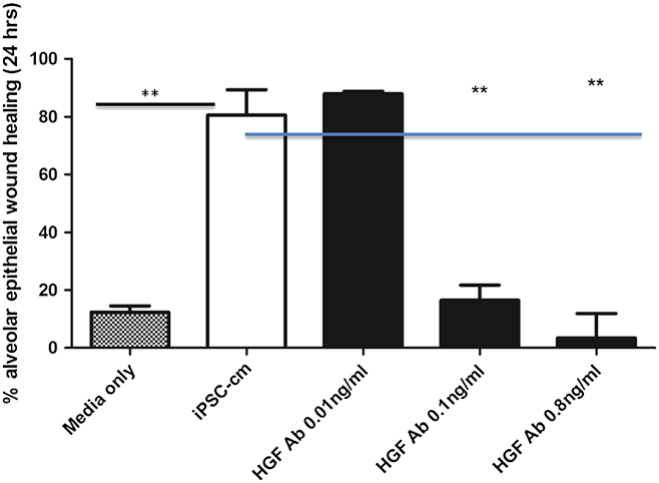
**Neutralizing anti-hepatocyte growth factor antibodies inhibit induced pluripotent stem cell-induced alveolar epithelial repair** ***in vitro*** **.** In the presence of neutralizing anti-hepatocyte growth factor antibodies (HGF Ab), the induction of alveolar epithelial repair *in vitro* by induced pluripotent stem cell conditioned medium (iPSC-cm) was reduced in a dose-dependent manner using the concentrations of HGF Ab as indicated. Data presented as mean ± standard error of the mean, **P <0.001.

Additional experiments were performed to identify soluble mediators in iPSC-cm that may contribute to the antifibrotic effect of iPSC-cm. Based on our previous data on the antifibrotic effect of HGF [24], we hypothesized that part of the repair-inducing and antifibrotic activities of iPSC-cm may be due to HGF secreted by iPSCs. HGF was present in biologically relevant concentrations in iPSC-cm (399.23 ± 2.9 ng/ml) and specific inhibition of HGF using HGF neutralizing antibody resulted in a significant and concentration-dependent decrease of iPSC-induced alveolar epithelial wound repair *in vitro*, indicating a central role for HGF in the epithelial repair-inducing activities of iPSCs (80.6 ± 8.7% wound closure induced by iPSC-cm vs. 16.4 ± 5.3% closure in presence of 0.1 ng/ml and 3.5 ± 8.4% wound closure in presence of 0.8 ng/ml HGF neutralizing antibody; *P* <0.001) (Figure 4). These data indicate that HGF secreted by iPSCs plays a prominent role in iPSC-cm induced alveolar epithelial repair *in vitro*.

**Figure 5:**
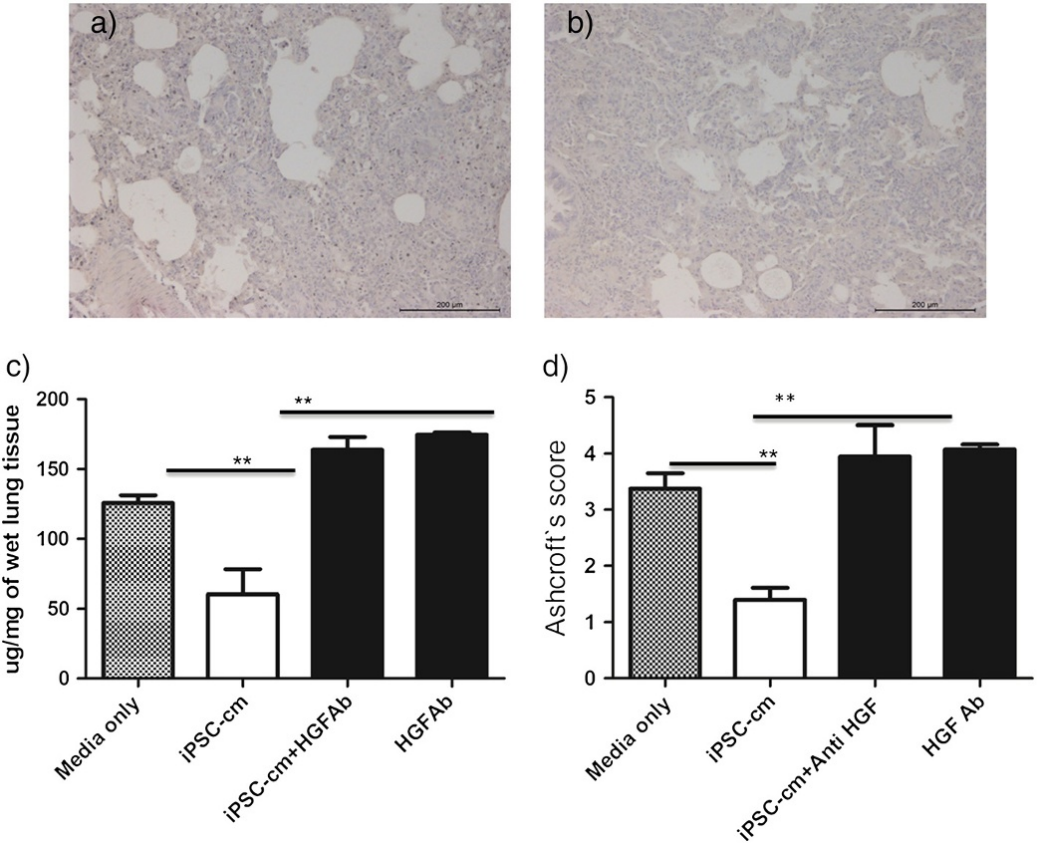
**Neutralizing anti-hepatocyte growth factor antibody inhibit induced pluripotent stem cell conditioned medium-induced reduction of lung fibrosis** ***in vivo*** **.** Induced pluripotent stem cell conditioned medium (iPSC-cm)-induced reduction in lung fibrosis was prevented by neutralizing anti-hepatocyte growth factor antibodies (HGF Ab) added to iPSC-cm prior to intratracheal instillation in the bleomycin-injured lung as assessed by **(a)** histology, **(c)** collagen content and **(d)** Ashcroft score in the lung. **(b)** When bleomycin-injured animals were treated with only HGF Ab there was no reduction in fibrosis, and the Ashcroft score and collagen content were significantly high. Data presented as mean ± standard error of the mean, **P <0.001).

To evaluate whether the observed antifibrotic effect of iPSC-cm *in vivo* is mediated by HGF, HGF neutralizing antibody was added to iPSC-cm before intratracheal administration 7 days after bleomycin lung injury. In the presence of HGF neutralizing antibody, the antifibrotic effect of iPSC-cm was abolished (Figure 5a). In accordance, the lung collagen content increased in the presence of HGF neutralizing antibody (60.5 ± 15.4 μg/mg after treatment with iPSC-cm vs. 164 ± 9.1 μg/mg wet lung tissue after treatment of iPSC-cm with anti-HGF antibody, *P* <0.001) (Figure 5c) and the Ashcroft score increased (1.40 ± 0.24 after iPSC-cm vs. 3.95 ± 0.6 after neutralizing with anti-HGF antibody, *P* <0.001) (Figure 5d). As controls, HGF neutralizing antibody alone did not show any antifibrotic effect in our *in vivo* model (Figure 5b); moreover, the total collagen content was increased (174.7 ± 1.76 μg/mg wet lung) and the Ashcroft score also worsened (4.07 ± 0.08, respectively).

### Myofibroblasts and TGFβ1 expression are reduced after treatment with iPSC-cm, in part by a HGF-dependent mechanism

**Figure 6:**
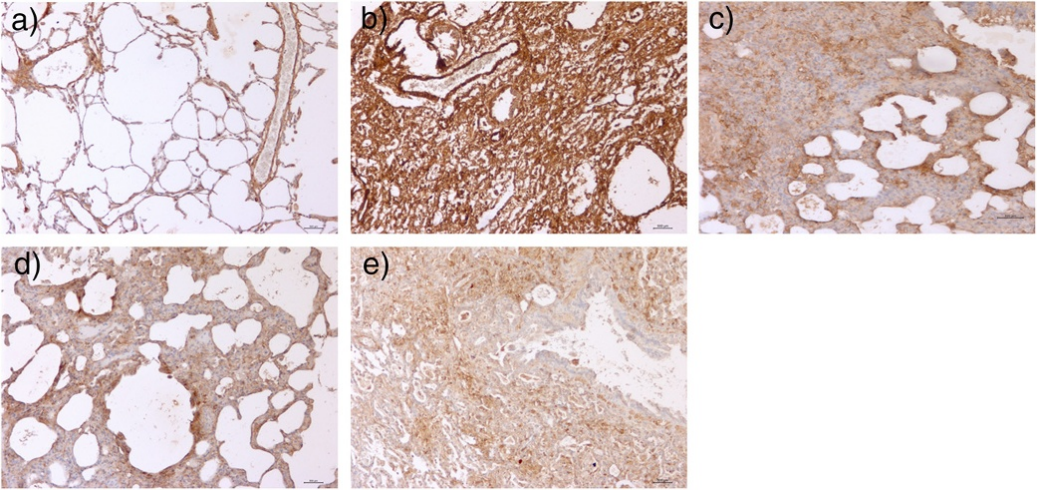
**Myofibroblast accumulation** ***in vivo*** **is reduced after treatment with induced pluripotent stem cell conditioned medium, in part by a hepatocyte growth factor-dependent mechanism.** Immunohistochemistry was performed to detect myofibroblasts using alpha smooth muscle actin (αSMA) antibody. In **(c)** media control and **(b)** fibroblast (CCD1) conditioned medium-treated animals, strong αSMA staining was detected; whereas in **(a)** induced pluripotent stem cell conditioned medium (iPSC-cm)-treated animals, the number of αSMA-positive signals was reduced. **(d)** When iPSC-cm was pretreated with neutralizing anti-hepatocyte growth factor (HGF) antibody before intratracheal instillation to bleomycin-injured lungs, αSMA positivity increased compared with treatment with iPSC-cm alone. **(e)** Treatment with HGF neutralizing anti-HGF alone did not show any effect compared with control animals. Scale bar =500 μm.

After treatment with iPSC-cm, cells positive for αSMA were markedly reduced in the bleomycin-induced lung injury model (Figure 6a) compared with the CCD1-cm-treated group (Figure 6b) or media control (Figure 6c), indicating a reduction of myofibroblasts in the injured and fibrotic areas of the lung. The expression of profibrotic TGFβ1 was reduced after treatment with iPSC-cm compared with control (0.9 ± 0.3 after treatment with iPSC-cm vs. 3.04 ± 0.4 (media control) and 3.5 ± 0.4 (CCD1-cm), expressed as relative gene expression changes (2 − ΔΔCt)). Treatment of bleomycin-injured lungs with iPSC-cm after addition of neutralizing anti-HGF antibodies resulted in an increase of TGFβ1 levels (1.85 ± 0.9, expressed as relative gene expression changes (2 − ΔΔCt)). In addition, the extent of myofibroblasts increased as shown with increased αSMA-positive cells after treatment with iPSC-cm treated with neutralizing anti-HGF antibodies (Figure 6d); treatment with HGF neutralizing antibody alone did not reduce αSMA-positive cells (Figure 6e). This data indicate that iPSCs secrete HGF that may be substantially involved in the antifibrotic effect of iPSC-cm observed *in vivo* in the bleomycin-induced lung injury and fibrosis model.

## Discussion

We show in this study that the secretome of iPSCs induces alveolar epithelial repair *in vitro* and reduces lung fibrosis *in vivo*, in part by a HGF-dependent mechanism. In particular, iPSC-cm showed improved alveolar epithelial wound repair properties in our *in vitro* lung epithelial wound healing model, and intratracheal instillation of iPSC-cm attenuates fibrosis in the bleomycin-injured rat lung, as shown by histology and reduced total collagen content. Moreover, high levels of HGF were identified in iPSC-cm, and in the presence of HGF neutralizing antibody the effect of iPSCs on alveolar epithelial wound repair *in vitro* and reduction of lung fibrosis in the bleomycin model was in part abolished. This data support our hypothesis that HGF secreted by iPSCs contribute to the antifibrotic effect induced by the secretome of iPSCs.

IPF is a devastating progressive lung disease characterized by pulmonary scar formation leading to respiratory failure and death. The prognosis is extremely poor, the exact pathophysiology is still unknown and there is so far no promising treatment available. However, recent evidence suggests that dysregulated alveolar repair processes contribute to the development of fibrosis in the lung [25]. Failure of the normal wound healing processes in response to repeated alveolar injuries thus led to an imbalance of the chemokines, cytokines, growth factors and enzymes such as proteinases and their inhibitors, leading to uncontrolled proliferation of fibroblasts, excess deposition of extracellular matrix and accumulation of myofibroblasts [26, 27]. A well regulated repair process in response to injury is essential to avoid the development of fibrosis. In recent times, cell-based therapies have been tried as a novel therapeutic option for fibrotic diseases, also in the lung [7, 28].

iPSCs have gained lot of attention in recent years due to their potential for regenerative medicine. Introduction of four genes to somatic cells leads to generation of iPSCs, which results in pluripotency comparable with embryonic stem cells [14, 21]. This technique has been tested in several species [29, 30] using different somatic cell types [29, 31–34], and has been used for rare genetic disorders in clinical settings [35, 36]. Some studies have shown beneficial effects of iPSCs in experimental animal models of various diseases yet the fate of implanted iPSCs in the diseased body is not yet described; moreover, there are concerns about malignant transformation of the iPSCs after application. Recent studies, however, showed the therapeutic potential of the conditioned media of stem cells in various disease models [37, 38]; the study by Gupta and colleagues showed in an *in vitro* co-culture model that mesenchymal stem cells secrete anti-inflammatory cytokines and decrease the tumor necrosis factor alpha release of macrophages in the acute lung injury model [39].

We hypothesized that iPSC-cm induces alveolar epithelial repair in an *in vitro* alveolar wound repair model and reduces pulmonary fibrosis in a bleomycin-induced lung injury and fibrosis model in the rat. A paper by How and colleagues published recently has reported therapeutic benefit of the iPSC secretome in lung injury [40]. In the present study, the results obtained with our *in vitro* alveolar epithelial wound repair assay and bleomycin-induced fibrosis model *in vivo* suggest that iPSC-cm may contain some biologically relevant secreted mediators which enhance alveolar epithelial repair and regeneration and reduce lung fibrosis *in vivo*.

HGF is an important growth factor and plays a major role in lung development in the early embryonic stages [41], specifically by regulating epithelial development and efficient branching morphogenesis [42], and also latter in the adult lung and in various disease processes including pulmonary fibrosis [43, 44]. Altered pro-HGF production by fibroblasts from IPF patients has been reported [45]. HGF is known to have beneficial effect when administered in the bleomycin-induced fibrosis models [46, 47]. In particular, we showed that targeted, electroporation-mediated expression of HGF specifically in alveolar type II epithelial cells results in a reduction of lung fibrosis in the bleomycin-induced lung fibrosis model [24]. High levels of HGF were detected in the iPSC-cm, and thus we hypothesized that the antifibrotic effect observed in the present study both in *vitro* and *in vivo* is partly mediated by HGF. Our hypothesis was supported by a series of inhibiting experiments where the alveolar epithelial repair activities of iPSC-cm treated with HGF neutralizing antibody was markedly diminished *in vitro* in a dose-dependent manner. Furthermore, the iPSC-cm pretreated with anti-HGF neutralizing antibody further aggravated the fibrotic process when instilled in the bleomycin-injured rat lungs; the total collagen content of the lung was increased and also the histological score showed aggravated fibrosis. These findings indicate that HGF present in the iPSC-cm reduces fibrosis and plays a crucial role in alveolar epithelial repair *in vitro* and contributes significantly to the antifibrotic properties of iPSC-cm.

TGFβ1, an important profibrotic mediator, was reduced in lung tissue homogenate after instillation of iPSC-cm in the bleomycin-injured rat lungs compared with controls. Interestingly, when iPSC-cm treated with HGF neutralizing antibody was instilled, TGFβ1 levels increased drastically in the lung tissue homogenate. αSMA was markedly reduced in the interstitial space after iPSC-cm treatment as observed by immunohistochemistry, indicating a substantial reduction of myofibroblasts in the fibrotic lung tissue after iPSC-cm treatment as reported recently [40], supporting the antifibrotic effect induced by iPSC-cm in our *in vivo* model. However, our study differs in many aspects. In the study by How and colleagues, iPSC-cm was administered 24 hours after bleomycin challenge and analysis was performed at day 3 post treatment [40], which is an acute model with high inflammatory component. We performed the iPSC-cm treatment 7 days after bleomycin administration, and performed analysis 7 days after iPSC-cm treatment (14 days after bleomycin administration). We demonstrate the beneficial effect of iPSC-cm in a more chronic model of bleomycin-injured rat lung, where the fibrotic component overwhelms the acute inflammatory response to bleomycin.

Interestingly enough, the treatment of iPSC-cm is very effective in the bleomycin-injured rat lungs, indicating that the secreted mediators have a strong therapeutic effect and can be a good substitute for cell therapy. However, the detailed analysis of the iPSC-cm is required and also its long-term effect needs to be evaluated. Most interestingly, iPSCs offer the possibility to generate patient-tailored therapy when the patient’s own somatic cells can be reprogrammed to iPSCs and used for treatment, thus circumventing the immune response that is usually associated with therapies using cells from different donors. Using iPSC-cm from patient-specific iPSCs will therefore offer a more promising, safe and effective therapeutic option.

In the current study we generated iPSCs using the pSIN vectors (nonreplicating lentiviral vectors) that are not expressed in the reprogrammed cells [48], yet the concern of using the viral vector cannot be ignored. New nonviral methods for generation of iPSCs have been reported recently using mRNA [49, 50] and recombinant protein [51]; since the iPSC is a new and evolving technology it will take some time and effort to modify the existing protocols, but enormous strides have been already made in the field aiming to reduce the use of xeno products for iPSC generation and culture [52]. Considering these technical advances in the future, the conditioned media obtained from iPSCs, generated using nonviral methods and cultured in xeno-free conditions, might make clinical application easier. Another concern is the method to produce large amounts of iPSC-cm; some efforts have been made in this direction [53], but a compact and efficient bioreactor needs to be engineered to meet the clinical criteria.

In summary, we show for the first time that iPSC-cm induces alveolar repair *in vitro* and reduces bleomycin-induced lung injury and fibrosis *in vivo*. Although further studies need to be performed, we assume that improvement of alveolar epithelial repair induced by iPSC-cm contributes significantly to the antifibrotic effect we observed after intratracheal iPSC-cm instillation. HGF obviously represents a crucial mediator in the secretome of iPSCs and contributes to the pro-repair and anti-fibrotic properties of iPSC. However, more detailed analysis of the secretome of iPSCs still needs to be elucidated.

## Conclusions

In the first proof-of-concept study we show that conditioned media obtained from iPSCs improved alveolar epithelial repair *in vitro* and attenuated bleomycin-induced lung injury and fibrosis *in vivo*, partially by a HGF-dependent mechanism. IPSC-cm therefore has therapeutic potential in lung fibrosis and might offer a promising novel option for patients suffering from pulmonary fibrosis, taking advantage of a novel cell-based, yet cell-free therapy based on the generation of iPSCs.

## Electronic supplementary material

Additional file 1: Figure S1: Showing cells stained for Annexin/PI. Fluorescence-activated cell sorting analysis was performed. The positive cells were compared with unstained cells and percentage of Annexin/PI-positive cells was calculated. There was no difference between cells growing in serum replacement and bFGF-free media and cells growing in normal media. (TIFF 849 KB)

## Abbreviations

bFGF: β-fibroblast growth factor
CCD1-cm: fibroblast conditioned medium
HGF: hepatocyte growth factor
IPF: idiopathic pulmonary fibrosis
iPSC: induced pluripotent stem cell
iPSC-cm: induced pluripotent stem cell conditioned medium
PBS: phosphate-buffered saline
PI: propidium iodide
TGFβ1: transforming growth factor beta 1
αSMA: alpha smooth muscle actin.
