# Supplementary figures and images for: The secretome of induced pluripotent stem cells reduces lung fibrosis in part by hepatocyte growth factor
Source: Stem Cell Res Ther. 2014 Nov 10;5(6):123. doi: 10.1186/scrt513 (PMC4445988; doi:10.1186/scrt513)

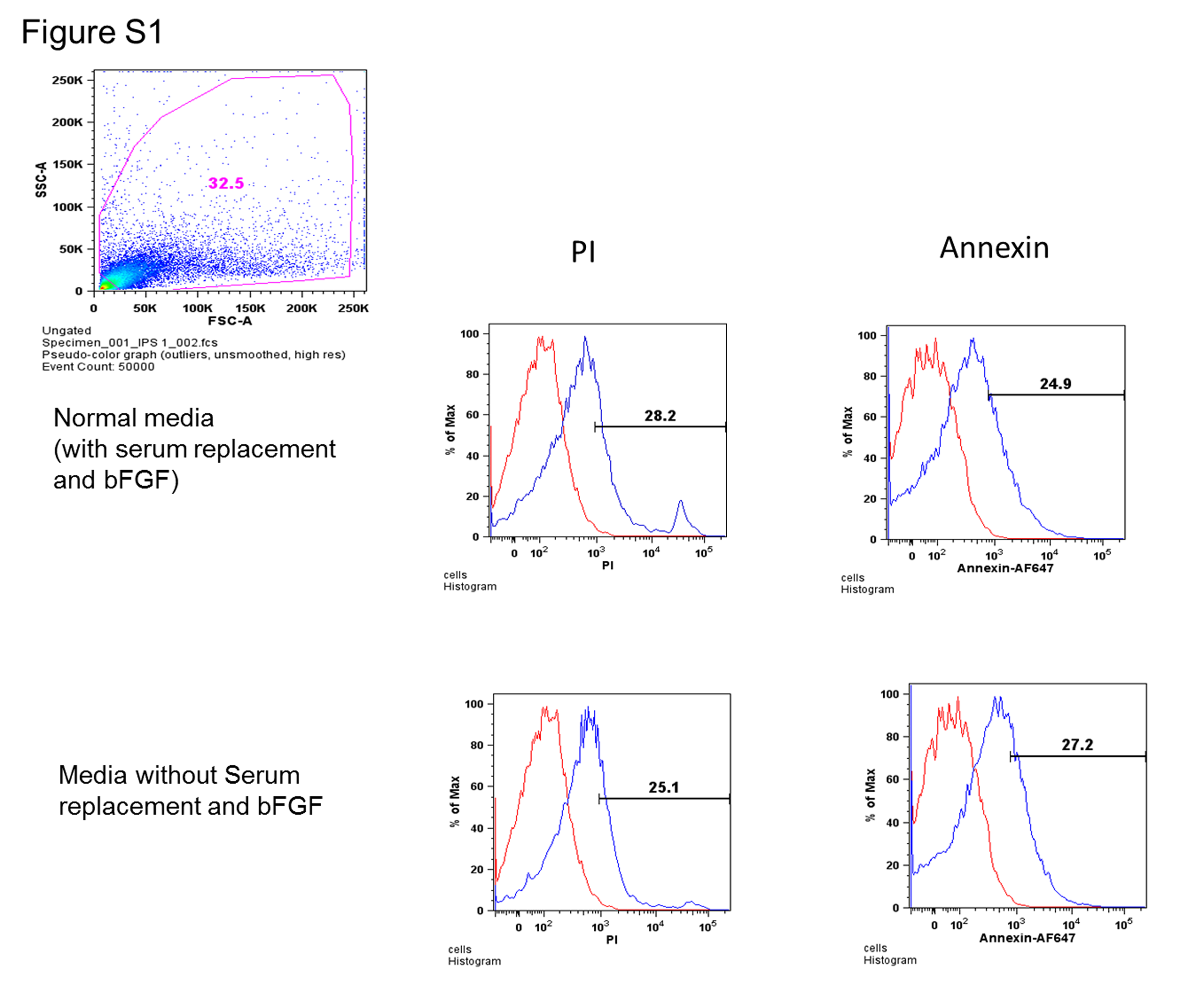

Supplement: Supplementary file 1 — Additional file 1: Figure S1: Showing cells stained for Annexin/PI. Fluorescence-activated cell sorting analysis was performed. The positive cells were compared with unstained cells and percentage of Annexin/PI-positive cells was calculated. There was no difference between cells growing in serum replacement and bFGF-free media and cells growing in normal media. (TIFF 849 KB) [file 13287_2014_435_MOESM1_ESM.tiff]
